# Disease response criteria in Langerhans cell histiocytosis: a global view

**DOI:** 10.1007/s12185-025-03989-z

**Published:** 2025-04-27

**Authors:** Ilia N. Buhtoiarov, Milen Minkov, Reza Vali, Oussama Abla

**Affiliations:** 1 Division of Pediatric Hematology, Oncology, and Blood and Marrow Transplantation, Pediatric Institute, Cleveland Clinic Foundation, 9500 Euclid Ave, R3-118, Cleveland, OH 44195 USA; 2 Department of Pediatric Hematology/Oncology, University Clinic of Pediatrics, Medical University of Vienna, Vienna, Austria; 3 Department of Diagnostic & Interventional Radiology, The Hospital for Sick Children, University of Toronto, Toronto, ON Canada; 4 Division of Hematology/Oncology, Department of Pediatrics, The Hospital for Sick Children, Toronto, ON Canada

**Keywords:** Langerhans cell histiocytosis, Therapy, Response, Harmonization

## Abstract

Langerhans cell histiocytosis (LCH) is an inflammatory myeloid neoplasm with heterogeneous presentations. The discovery of BRAFV600E and other MAPK pathway mutations drastically transformed the treatment landscape, especially for high-risk LCH and CNS-LCH. While treatment strategies for children and adults are somewhat similar, response assessment methodologies remain highly dichotomized.

Currently, separate treatment response criteria exist for children and adults, especially in therapeutic trials. Considering the rapid evolution of targeted MAPK-inhibitor therapies, along with ultrasensitive detection of minimal residual disease biomarkers (e.g., circulating BRAFV600E-encoding DNA) and sophisticated imaging tools (18F-FDG-PET and whole-body MRI), harmonization of response criteria in LCH is clearly warranted. The Histiocyte Society Global LCH Treatment Response Harmonization Task Force, a collaborative network of pediatric and adult LCH experts, is set to propose updated pediatric LCH treatment response criteria, which will also serve as the foundation for a universal response assessment tool for pediatric and adult LCH. In this review, we focus on the past, present, and likely future of response assessment in LCH patients, and discuss needs that remain unmet in the targeted therapy era.

## Introduction

Langerhans cell histiocytosis is a rare inflammatory neoplasm of myeloid origin characterized by the presence of classic CD1A^+^CD207^+^ histiocytes notorious for aberrant function secondary to activating somatic mutations in the MAPK (RAS–RAF–MEK–ERK) pathway. The most common somatic mutation is BRAF-V600E, which can be found in more than 60% of patients. Irrespective of the type of mutually excluding mutations in these signaling proteins, the Langerhans cells persist in the permanently activated state due to constitutively activated MAPK pathway [1].

LCH is known to affect both children and adults, and yet is more commonly seen in the young pediatric population [2, 3]. In about half of the patients, LCH presents with multisystem (MS) involvement, while the remaining patients present with single-system (SS) disease, affecting mostly bones, skin, or lungs [4, 5]. Recent research discoveries revealed that while the CD1A^+^CD207^+^ histiocytes are the hallmark of LCH, other BRAF-mutated peripheral blood mononuclear cell populations (such as NK cells and granulocytes) may contribute to the substantial diversity of LCH clinical manifestations.

Current advances in the treatment of childhood LCH have been paved by clinical trials conducted by the Histiocyte Society, the international non-profit professional organization aiming to advance knowledge and improve outcomes for patients with histiocytic disorders through clinical and basic research and education.

## The past

The first classification of disease response assessment in childhood LCH was established by the International LCH Study Group [6, 7]. The terms “Non-Active disease [NAD]”, “Active Disease [AD]—better”, “[AD]—Stable disease”, and “[AD]—Progressive Disease” (Table 1) were used in the LCH-I (1991–1995), LCH-II (1996–2001), and LCH-III (2001–2008) clinical trials [8–10]. In the LCH-III trial, treatment response in MS-LCH patients with high-risk organ (RO^+^; liver, spleen, and bone marrow) involvement was assessed by changes in those organs but not in other “low-risk” sites like bones, skin, lymph nodes, or lungs. On the other hand, the treatment response assessment in RO^−^ MS-LCH patients was based on regression of the lesions in the involved organ systems [10]. The role of conventional imaging in disease response assessment in these LCH clinical trials relied on radiographic skeletal surveys and computed tomography (CT) scans, while the use of [18F]Fluorodeoxyglucose positron emission tomography (^18^F-FDG-PET) scans or the whole-body magnetic resonance imaging (WB-MRI) was not adopted.

**Table 1:** Disease activity state and the treatment response categories for pediatric LCH

Main category	Sub-categories	Definition
Non-active disease (NAD)		Resolution of all signs and/or symptoms No evidence of disease
Active disease (AD)		
Better	Regressive disease	Regression of signs and/or symptoms, no new lesions
Stable	Stable disease	Persistence of signs and/or symptoms no new lesions
Worse	Progressive disease*	Progression of signs and/or symptoms Appearance of new lesions

Response category	Definition
Better	Complete resolution (NAD) Regression (AD better)
Intermediate	Stable (unchanged)
Worse	Progression*

^*^Progression of skeletal lesions is defined as unequivocal enlargement of the size of existing lesions and/or appearance of new lesions. In patients with risk organ involvement, the overall response depends on response in risk organs

Similarly, in JLSG-96 (1996–2001) and JLSG-02 (2002–2009) clinical trials, conducted by the Japan LCH Study Group, the disease response assessment was categorized as good response (equivalent to NAD), partial response (regression of > 50% of signs and symptoms), non-response (regression of < 50%), and progressive disease. Conventional imaging was limited to skeletal surveys to assess skeletal lesions, ultrasonography to assess liver and spleen size, and the high-resolution CT for pulmonary LCH, while ^18^F-FDG-PET scans were not adopted either [11, 12].

In an attempt to overcome the limitations of such semi-quantitative disease response assessment, Jean Donadieu et al. proposed an LCH disease activity scoring (DAS) system that relied on measuring the magnitude of signs and symptoms associated with major organ system involvement (Table 2) [13]. This semi-quantitative scoring system could help predict the pediatric LCH patient survival based on constellation of radiographic changes as well as clinical symptoms assigned a score from 0 to 5 based on their magnitude and extend at the time of diagnosis, as well as this combined score changes at the time of first assessment at 6 weeks of therapy. This DAS system allows for upfront identification of the cohort of patients, invariably with a higher score (≥ 7) at the time of diagnosis, who are likely to progress on therapy, or experience disease re-activation following therapy completion. This assessment algorithm extended beyond the conventional classification of LCH as low-, standard-, and high-risk disease, which is otherwise used in the most recent clinical trials (LCH-III and LCH-IV) for the outcome assessment. Those patients, whose score increased by more than 1 point while of therapy, either due to involvement of a new organ system, or due to worsening of the clinico-radiographic picture, had unfavorable outcome. And while this elegant scoring system is versatile and highly predictive of the treatment outcome, it is not without the limitations. While it included augmented scoring provisions for the hematological system involvement, it scored liver and spleen involvement (the high-risk organs) similar to musculo-skeletal and skin LCH (the low-risk conditions). In addition, this DAS clinical assessment system did not include any scoring provisions for CNS-LCH, which can manifest as both LCH-associated abnormal CNS imaging (LACI) and LCH-associated abnormal CNS symptoms, with the later commonly being the functional consequence of advanced LACI [14]. Furthermore, this DAS scoring system was not well fitted for the assessment of bone lesions in the appendicular skeleton and skull, the commonest involved system in children and adults; it relied only on the assessment of signs and symptoms, and not on any radiographic changes [2, 15]. Also, the DAS system assigned the highest score (up to 5) for the pulmonary LCH (standard-risk organ), which commonly could be a reason for acute morbidity, but very rarely the LCH-related mortality. Pneumothorax, as one of the non-specific radiographic manifestations of pulmonary LCH, has been assigned a high score of 2, whereas it is rather a secondary event associated with cystic transformation of parenchymal granulomas in the subpleural space, while the densities of the early granulomatous lesions are related to severity of respiratory distress. In addition, changes in the respiratory performance (improvement vs deterioration) status do not directly correlate with trajectory and dynamics of changes in the major parameters of the pulmonary function testing. At last, the pulmonary function testing has suboptimal degree of reproducibility in patients of younger age, or may require additional tools for a proper assessment of the abnormal pulmonary function [16–18].

**Table 2:** The clinical score for LCH disease activity

Variable	Modality	Score
Bone (a)	Pain	1
	No pain	0
Bone (b)	Compressing other organs (orbit or spine)	2
	No compression	0
Fever (> 35.5C)	Yes	1
	No	0
Lungs: iconography	Pneumothorax	2
	Interstitial lesion on chest X-ray or lung CT scan	1
	Normal chest X-ray or chest CT scan	0
Lung: function	Mechanical ventilation and PFT > 50%	5
	Supplemental oxygen or PFT between 50 and 80%	2
	No dysfunction, no cyanosis, no supplemental oxygen	0
Skin: area	> 25%	2
	5–25%	1
	< 5%	0
Soft-tissue tumor, including CNS	> 5 cm max diameter	2
	2–5 cm max diameter	1
	0–2 max diameter	0
Nodules (> 2 cm)	Yes	1
	No	0
Liver	Below umbilicus	2
	Enlarged above umbilicus	1
	Not enlarged	0
Liver enzymes	> 10 UNL	2
	3–10 UNL	1
	< 3 UNL	0
Liver gGT	> 10 UNL	2
	3–10 UNL	1
	< 3 UNL	0
Albumin	Albumin transfusion required in past week	3
	< 3 g/dL	1
	> 3 g dL	0
Spleen	Below umbilicus	2
	Enlarged above umbilicus	1
	Not enlarged	0
Platelets: requirements in past week	> 2	4
	1–2	3
	Low count, but no platelets transfusion	2
	Normal count	0
RBC: requirements in past week	> 2	4
	1–2	3
	Low count, but no platelets transfusion	1
	Normal count	0

Verifying the bone marrow (BM) involvement with LCH has been and remains to be the major challenge. Multiple attempts have been made to establish the parameters of BM LCH. For pediatric LCH, the first set of parameters was proposed in 1975 by Lahey et al., based on the presence of anemia [hemoglobin < 10 g/dL (< 9 g/dL for infants)] and cytopenias (platelets < 100 × 10^6^/mL, white blood cell count < 4 × 10^6^/mL, and/or neutrophils < 1.5 × 10^6^/mL) [19] In the literature, it is commonly referred to as involvement of hematological system, thereby making the distinction between reactive changes, secondary hemophagocytosis, and concurrent myeloid malignancies practically invisible. Attempts to find morphological evidence of presence CD1a^+^ histiocytes revealed extreme rarity of those in BM: less than 10–20 cells/BM aspirate smear slide by immunohistochemistry, or less than 0.5% of all mononuclear cells by multicolor flow cytometry [20]. In the Donadieu’s DAS system, the main accept was made on transfusion dependence, based on the number of units of platelets or packed red blood cells transfused within a week [13].

While the Jean Donadieu’s DAS method elegantly extrapolated the extend of anatomical and functional changes, and the degree of symptoms into the numbers enabling to predict the likely outcome, this approach seemed to only partially address the matter of the actual clinical severity of LCH, which should be considerably higher in the patients with liver, spleen, and hematological system involvement, to more accurately reflect the morbidity patterns and clinical response trends in the patients with the high-risk multisystem LCH.

Until 2012, there were no universally accepted guidelines for the management and response assessment for adult LCH patients [15, 21]. Based on the retrospective analyses of national registries, the risk organs for adult LCH patients included bone marrow, liver, spleen, and CNS. Due to the lack of standard front-line therapies for adult LCH, disease response assessment after 2–3 cycles of therapy commonly relied on the same approaches used during initial diagnostic work-up [15].

## The present

Significant changes in clinical assessment practices have recently occurred in both pediatric and adult LCH patients, although these practices remain significantly dichotomized. The currently open LCH-IV trial for pediatric LCH (2013-present) provides precise guidelines on sonographic assessment of liver and spleen, yet primarily for changes in size. For skeletal lesions, the pre-therapy disease extent as well as treatment response assessment continue to rely on skeletal surveys, CT, and MRI. Further, ^18^F-FDG-PET became allowed as the pre-treatment evaluation method, but still would not be used as the sole method for the bone disease assessment, to avoid misinterpretation of the focal hypermetabolic inflammatory bone marrow activity as the overt bone lesions; only radiographically confirmed (by X-ray, CT, or MRI) destructive bone processes will be counted toward skeletal involvement. Meanwhile, the recently updated adult LCH management guidelines strongly support the use of ^18^F-FDG-PET as well as CT and MRI for the organ-specific assessment, both at the baseline and post-treatment [22]. In addition, LCH experts of the National Comprehensive Cancer Network (NCCN) in 2019 put together the clinical practice guidelines for pediatric and adult histiocytic neoplasms. These guidelines also recommend ^18^F-FDG-PET assisted by other organ-specific imaging tools for the initial diagnosis, response assessment, and post-treatment follow-up [23].

Despite the high versatility of available anatomical imaging techniques, they are not without the practical limitations. For example, conventional radiography is best used for assessment of lesions of calvarial bones, large bones of axial skeleton, and the long bones of appendicular skeleton. At the same time, presence of the soft-tissue component, as well as imaging of vertebral bodies and bones of viscerocranium, temporal bone, middle ear, and clivus may require concurrent application of CT and MRI due to need for a more accurate delineation of dense anatomical details. Under certain clinical circumstances, plain radiograph fails to distinguish new, active lesions from old, inactive lesions, thereby posing a challenging question related to clinically silent disease re-activation [24].

Hence, the momentum for re-validation of the role of these imaging tools and response assessment methodologies in the management of pediatric LCH patients has arrived, and a revised concept, with a high rate of sensitivity for changes in all organ systems for LCH patients with MS disease, is strongly warranted (Tables 3, 4).

**Table 3:** Treatment response criteria for adult LCH patients

Response category	Radiographic response criteria (18FDG-PET)	Radiographic response criteria (CT or MRI)
Complete response (CR)	Normalization of lesions with FDG uptake equal to surrounding background tissue	Complete anatomic resolution of lesions, or resolution of abnormal imaging features (enhancement, diffusion restriction, etc.)
Partial response (PR)	Reduction from baseline SUV of lesions, but persistent uptake greater than surrounding background tissue	Reduction, but not complete resolution of lesions/abnormal imaging features
Progressive disease (PD)	Increased SUV value of lesions as compared with before or appearance of new FDG avid lesions	Worsening of abnormal imaging features or growth of existing lesions or appearance of new lesions
Stable disease (SD)	Does not meet other criteria	Does not meet other criteria

*SUV* standardized uptake value

**Table 4:** The NCCN recommendations for the imaging-based evaluations of pediatric and adult LCH patients

Initial diagnostic evaluation	Treatment response assessment	Surveillance
*Baseline*		
Whole-body 18FDG-PETCT	18FDG-PETCT [preferred]	18FDG-PETCT scan (preferred)
	CT/MRI	CT/MRI
	After 2–3 cycles of therapy & at completion	
*Useful under certain circumstances, based on symptoms or organ involvement*		
High-resolution chest CT for suspected PLCH		
CT of the chest, abdomen, and pelvis with contrast		
MRI ± contrast for the following		
brain		
spine		
sella turcica ± pituitary (if DI suspected)		
USG of the abdomen for liver & spleen		
ERCP		
Digital panoramic X-ray		

*ERCP* Endoscopic retrograde cholangiopancreatography, *USG* ultrasonography, *DI* diabetes insipidus, *PLCH* pulmonary LCH

A wide spectrum of imaging manifestations of LCH lesions in different organs stems from the dynamics of evolution of pathological changes, from a granulomatous inflammatory infiltrate through the tissue destruction to the tissue healing through remodeling and scarring. In some instances, the early granulomatous lesions can regress without tissue destruction and subsequent scarring. The dynamics and trajectory of these involution changes varies in different organ systems, is affected by the factors inducing these changes, and has different timeline of detection by different imaging methods. For example, the earliest evidence of bone remodeling and healing on X-ray-based skeletal survey can be seen by 6 weeks, along with complete cessation of metabolic activity of granulomatous lesions on ^18^F-FDG-PET [25, 26]. The high sensitivity of ^18^F-FDG-PET for LCH lesions stems from accumulation of ^18^F-FDG contrast in the sites of hypermetabolic granulomatous inflammatory lesions. The whole-body ^18^F-FDG-PET scans can detect LCH activity, measure early response to therapy, and distinguish active from non-active LCH lesions with greater accuracy than any other imaging modalities in patients with LCH lesions in the bones and soft tissues.

Multiple *retrospective* studies have compared the sensitivity and specificity of various imaging modalities in LCH patients. The whole-body MRI has significantly higher sensitivity over other conventional imaging tools such as skeletal surveys or technetium bone scans in detecting bone lesions and accurate disease staging [27–29]. One possible downside of this imaging tool is that it may require procedural sedation for the young age children who may not stay still during this lengthy (up to 60 min) imaging procedure [30]. Further, whole-body ^18^F-FDG-PET/CT or ^18^F-FDG-PET/MRI have very low false-positive rates and appear to exceed WB-MRI, skeletal surveys, or bone scans in detecting more skeletal and extra-skeletal lesions, except for the lungs, liver, and CNS [24, 31, 32]. Importantly, ^18^F-FDG-PET/CT or ^18^F-FDG-PET/MRI can detect treatment-related changes much earlier and with greater accuracy than conventional imaging, similar to other pediatric tumors [31, 33, 34]. The currently opened phase 2 clinical trial of pan-RAF inhibitor Tovorafenib (DAY101) in relapsed and refractory Langerhans Cell Histiocytosis led by the Children’s Oncology Group (COG ANHL2131; NCT05828069) will provide the opportunity for *prospective* comparison of sensitivity and specificity of these imaging methods as one of this study’s exploratory objectives.

The PET Response Criteria in Solid Tumors (PERCIST) were created in 2009 to ensure a systematic and structured assessment of treatment response with ^18^F-FDG-PET/CT [35]. Measurement of the most metabolically active lesions, and the background, metabolically inert areas on imaging (the right lobe of the liver and the mediastinal blood pool) are the PET imaging baseline principles. This approach can guarantee that ^18^F-FDG-PET is carried out properly, and that comparison between tests performed at different time points is feasible [36].

However, LCH is not a solid tumor, and it remains controversial whether PERCIST criteria can be adopted for LCH patients. While multiple studies addressing the utility of ^18^F-FDG-PET in LCH demonstrated adherence to PERCIST methodological principles [31, 37–39], there are instances where the surrounding tissue was used as the metabolic background reference standard [22, 40]. Further, treatment response categories on ^18^F-FDG-PET scans (complete metabolic remission [CMR]; partial metabolic remission [PMR]; stable metabolic disease [SMD], and progressive metabolic disease [PMD]) do not align well with the principles used by the LCH-IV trial (NAD, AD better, intermediate, worse).

Treatment effects (complete remission [CR]; partial remission [PR]; stable disease [SD], and progressive disease [PD]) evaluated by anatomical imaging (CT or MRI) according to the Response Evaluation Criteria in Solid Tumors (RECIST) may define changes as CR status when the metabolically active lesions as assessed by PERCIST criteria could still be present [39]. Similarly, lesions that might be considered as PR on anatomical imaging (CT) may also include still metabolically active disease if PERCIST principles are simultaneously applied [41]. Therefore, CR status by RECIST criteria may be equal to CMR or PMR on PERCIST scoring. The fact that some early granulomatous lesions, i.e., before the tissue destruction, can only be detected by ^18^F-FDG-PET and not by anatomical imaging may indicate that RECIST criteria are not of universal practical utility in LCH [39, 42]. Similar scenario was reported in patients with CNS-LCH, where changes seen by ^18^F-FDG-PET may precede neuroradiographic abnormalities detected by MRI [43]. The usefulness of ^18^F-FDG-PET for CNS-LCH remains limited due to high background metabolic activity of the brain. Of interest, the neurodegenerative CNS-LCH presents as hypometabolic changes on ^18^F-FDG-PET which is likely secondary to the neuronal loss.

In recent years, strong attention has been drawn to the prognostic significance of the circulating mutation copies encoding BRAF-V600E (cDNA^V600E^), both cell-free DNA^V600E^ as well as mutant DNA^V600E^ alleles harbored in the peripheral blood mononuclear cells, in the disease treatment response assessment and prediction of outcome. *BRAF*^V600E^ is the commonest activating mutation accounting for ~ 60% of disease and is uniquely predominant in patients with multisystem LCH involving risk organs and LCH-associated neurodegeneration. Among other laboratory biomarkers that can help predict LCH activity in various organ systems, such as alkaline phosphatase, IL-17, TNF-α, receptor activator of NF-κB (RANK) and its soluble ligand RANKL, sclerostin, periostin, osteopontin, osteoprotegerin, and neurofilament light-chain protein (NLP), cDNA^V600E^, without doubts, is the most studied one.[44, 45] In pediatric patients, the cell-free DNA^V600E^ can be detected more frequently than PBMC-DNA^V600E^; patients who have both cell-free DNA^V600E^ and PBMC-DNA^V600E^ are likely to be more symptomatic from their disease and have a higher DAS score. One significant factor limiting enthusiasm about this analyte is that cDNA^V600E^ can be found only in the fraction of patients with LCH harboring DNA^V600E^ in the tumor, predominantly younger children with multisystem LCH and the risk organ (liver, spleen, and hematological system) involvement, thereby restricting its practical applicability in LCH patients who have other than BRAF-V600E mutation genetic alterations within MAPK pathway [46–51].

And yet, a strong correlation between persistence of cDNA^V600E^ with clinical response at 6 weeks of therapy and with the rate of disease re-activation was observed [52]. However, it remains to be determined how changes in cDNA^V600E^ load would correlate with treatment effects seen on the conventional imaging, and how those changes could guide subsequent treatment strategies [53]. In other words, at present time, it seems to be premature to recommend this tool for universal use as an additional reliable biomarker of disease response, and more studies are needed.

Neurofilament light‐chain protein (NLP) in cerebrospinal fluid (CSF) is a sensitive and well‐established universal biomarker of neuroaxonal damage, including in LCH patients. Elevated plasma NLP concentration > 2 ng/mL was suggested as a sensitive screening tool for ND-CNS-LCH, even in clinically asymptomatic patients. In addition, serial monitoring of plasma NLP was shown to be of practical value for monitoring of the effect of therapy in ND‐CNS‐LCH, although the predictive power of this biomarker on the strength of treatment response remains to be understood [54–56].

Osteopontin is another useful CSF protein commonly seen elevated in patients with CNS-LCH compared with non-LCH patients with other degenerative brain pathologies. At the same time, the presence of cDNA^V600E^ in CSF is rather the infrequent occurrence [57].

## The future

The optimal approach toward treatment response assessment for children with LCH remains the major unmet need. The Histiocyte community is slowly gravitating toward the use of more sophisticated tools, such as ^18^F-FDG-PET and WB-MRI, especially in the context of research clinical trials; however, these imaging tools may not be equally accessible or affordable in all regions of the world. Of note, the majority of pediatric LCH patients are treated outside of clinical trials. In addition, the liberal application of ^18^F-FDG-PET for staging, response monitoring, and post-therapy follow-up inflicts substantial radiation exposure, especially in the case of multiple scans, and financial hardship for patients from the low-income countries [58] ^18^F-FDG-PET is the least accessible diagnostic imaging modality for patients outside North America, Western Europe, Japan, and Australia [59]. Furthermore, WB-MRI and ^18^F-FDG-PET scans usually require procedural sedation in infants and young children to avoid motion artifacts, and this is another major limitation for children with LCH. Additional barriers to overcome in the low-resource settings of the”real world” are the high cost of PET scanners; cost associated with the scanners maintenance and calibration; cost of nuclear medicine and radiology staff and biomedical engineers; availability of the cyclotron units for ^18^F-FDG production; guidelines for clinically appropriate and the cost-effective use of the functional imaging for LCH and other cancers; adherence to the nuclear safety regulations [60]. The true cost-effectiveness of use of ^18^F-FDG-PET for patients with LCH and other histiocytic neoplasms remains to be demonstrated. However, the opportunity to assess the early response to the treatment, as well as early disease re-activation by changes in inflammatory metabolic activity within lesions adds to the practical value of this imaging tool [32, 33, 42]. Establishment of so-called regional centers of reference for a better utilization of ^18^F-FDG-PET could help address many of the aforementioned concerns; yet, it would imply the existence of infrastructure and logistics to support the patients’ (sometimes frail and fragile) short- or long-distance travel [61].

It also remains to be validated into clinical practice how the kinetics of cDNA^V600E^ copies in the peripheral blood may correlate with the reduction of metabolic activity of LCH lesions on ^18^F-FDG-PET scan, especially in the context of MS-LCH involving risk organs. The lessons learned from patients treated with conventional chemotherapy (vinblastine and prednisone) may not be fully applicable to patients treated with BRAF or MEK inhibitors, or those patients who might be treated with chemoimmunotherapy. There may be lack of direct correlation between the cDNA^V600E^ measurement and clinical response in patients treated with inhibitors or conventional chemotherapy, or a combination of two. Of note, cDNA^V600E^ is detectable only in a faction of patients, mostly in the high-risk MS-LCH settings, which adds practical value to the harmonization efforts put forward by the LCH Treatment Response Harmonization Task Force.

At the present time, there is no uniform standard of how to most efficiently and cost-effectively assess the treatment response in patients treated with MAPKi, with or without chemotherapy. While some clinical researchers continue to rely of DAS system, other uses the more advanced clinico-radiographic methods and molecular tools to obtain a more granular information on the treatment-induced changes depending upon research questions [62–66].

NCCN clinical practice guidelines for histiocytic neoplasms remain to be one of the greatest free reference resources around the world. In 2024, there was a total of 18,972 downloads of the NCCN clinical practice guidelines for histiocytic neoplasms across 152 countries, as recorded by the NCCN headquarters. In February of 2025, the experts of the histiocytic neoplasms panel met to review the detailed proposal of pediatric LCH-specific guidelines, which is currently under review. The proposal uniquely focuses on the safe and cost-effective methods of initial diagnostic evaluation, in-therapy assessment, and post-therapy follow-up, as well as practice-proven methods of therapy. And whereas the panel experts are the members of the US medical institutions only (32 total), the NCCN clinical practice guidelines are uniquely meant for global use.

The widespread introduction of the immune checkpoint inhibitors and other immunotherapies into oncology practice triggered a rigorous revision of both RECIST and PERCIST criteria, to allow responses not typically observed in the traditional chemotherapy-based regimens to be identified and better documented [67, 68]. As the role of the PD-L1 blockade in LCH biology is being currently tested, it is quite likely that these revised immune-related RECIST (iRECIST) and immune-related PERCIST (iPERCIST) guidelines may also need to be validated for the treatment response assessment in LCH patients as well [69, 70].

The Histiocyte Society Global LCH Treatment Response Harmonization Task Force, consisting of LCH experts from North America (USA, Canada) and Europe (Austria, Sweden, Italy, and Greece), was established in 2023, with the aim of updating pediatric LCH treatment response criteria, understanding that one must be cognizant when using RECSIT or PERCIST criteria in LCH patients, as LCH is not a solid tumor but a chronic inflammatory myeloid neoplasm with no uniform imaging features in different organ systems. At the 40th Annual Histiocyte Society meeting in 2024, the LCH Treatment Response Harmonization Task Force leads presented the first detailed analysis of existing treatment response assessment practices, and proposed a systematic clinico-radiographic approach to the treatment response assessment for LCH involving skin, musculo-skeletal system, lymph nodes, thymus, lungs, liver, spleen, and bone marrow. The collaborative work is now in progress to bring to the practical balance the benefits of various clinico-radiographic, functional and molecular treatment response assessment tools, risks of radiation exposure, the need for deep sedation, their costs, and their real-world availability. Expert opinions from the resource-limited regions of the world play critical role for giving the harmonized response criteria the universal shape.

There is a unanimous agreement that the response assessment strategies will strongly benefit from a synergistic approach across all imaging modalities, including X-ray, ultrasound, CT, ^18^F-FDG-PET, and MRI of all disease sites, which can be applicable globally even in countries with limited resources.

## In conclusion

Explosive advances in understanding of LCH biology allowed for rapid evolution of therapeutic options. Recent reclassification of LCH as the neoplasm prompted rapid development of several management guidelines which, while are based on existing principles for adult cancer patients, do not fully embrace the unique pathophysiology of LCH as chronic inflammatory myeloid neoplastic process [16, 17, 71–73]. The practical incongruency between management recommendations for pediatric and adult LCH patients has therefore transformed into the impetus for harmonization of existing treatment response assessment paradigms, by redefining the imaging-based treatment response criteria and accommodating the newest molecular data of the circulating DNA^V600E^. As many patients continue to receive their therapy outside of the clinical trials, i.e., “as per” predecessor treatment plans, the results of treatment on newer protocol may be difficult to compare to the historic controls, especially when the methods of response assessment were dissimilar. The global collaboration on these challenges, under the auspices of the Histiocyte Society, through re-validation of old methods of treatment response assessment and introduction of the newer ones will further widen the perspective of improved outcomes for pediatric patients and adult with Langerhans cell histiocytosis.

## Abbreviations

AD: Active disease
BM: Bone marrow
cDNAV600E: Circulating DNAV600E
CMR: Complete metabolic remission
CNS: Central neuros system
COG: Children’s oncology group
CR: Complete remission
CSF: Cerebrospinal fluid
CT: Computed tomography
DNA: Deoxyribonucleic acid
LACI: LCH-associated abnormal CNS imaging
LACS: LCH-associated abnormal CNS symptoms
LCH: Langerhans cell histiocytosis
MAPK: Mitogen-activated protein kinase
MRI: Magnetic resonance tomography
MS: Multi-system
NAD: Non-active disease
NCCN: National comprehensive cancer network
NLP: Neurofilament light‐chain protein
PBMC: Peripheral blood mononuclear cells
PD: Progressive disease
PERCIST: Positron emission tomography response criteria in solid tumors
PD-L1: Programmed death ligand-1
PMD: Progressive metabolic disease
PMR: Partial metabolic remission
PR: Partial response
RECIST: Response evaluation criteria in solid tumors
SD: Stable disease
SMD: Stable metabolic disease
SS: Single-site
WB-MRI: Whole-body MRI
^18^F-FDG-PET: 18F-Fluorodeoxyglucose positron emission tomography
